# Effect of polar organic solvents on the separation of rare earths and transition metal chloride complexes: comparison of ion exchange, extraction chromatography and solvent extraction†

**DOI:** 10.1039/d5ra00908a

**Published:** 2025-06-03

**Authors:** Brecht Dewulf, Koen Binnemans

**Affiliations:** a Department of Chemistry, KU Leuven Celestijnenlaan 200F, P. O. Box 2404 B-3001 Leuven Belgium brecht.dewulf@kuleuven.be

## Abstract

In the search for more efficient purification and separation methods for rare earths, remarkable results were obtained in the field of solvometallurgy. Replacing the aqueous phase partially or largely by polar molecular organic solvents can significantly improve extraction efficiency and selectivity in the separation of rare earths and transition metals. The effect of polar organic solvents on the sorption of rare-earth elements and transition metals was investigated for strong anion exchanger Amberlite IRA 402 (Cl^−^), and for extraction chromatography resins, TEVA (Cl^−^) and DGA. The sorption of metal ions was tested from ethanol, ethylene glycol, water and formamide. While Amberlite IRA 402 selectively recovered the iron, copper and cobalt, while leaving the rare-earth elements in solution, the DGA resin was selective for rare-earth elements and could be used for the separation of heavy rare earths from light rare earths. The efficiency of metal uptake by the resins increased with decreasing dielectric constant of the feed solvent. These results were compared to non-aqueous solvent extraction using Aliquat 336 and TODGA from the different polar organic solvents. Significant mutual miscibility observed during these solvent extraction tests demonstrated the advantage of using ion-exchange resins over solvent extraction. However, in the case of chromatographic resins, the functional molecules are only physically bonded by the resin, and loss of sorption capacity due to extractant loss was observed with both TEVA and DGA resin, especially in combination with ethanolic solutions.

## Abstract 2

Ion exchangers are less prone to losses of extractant in polar organic solvents compared to extraction chromatography resins and solvent extraction systems, and yet retain the same selectivity towards transition metals and/or rare-earth elements.

## Graphical abstract

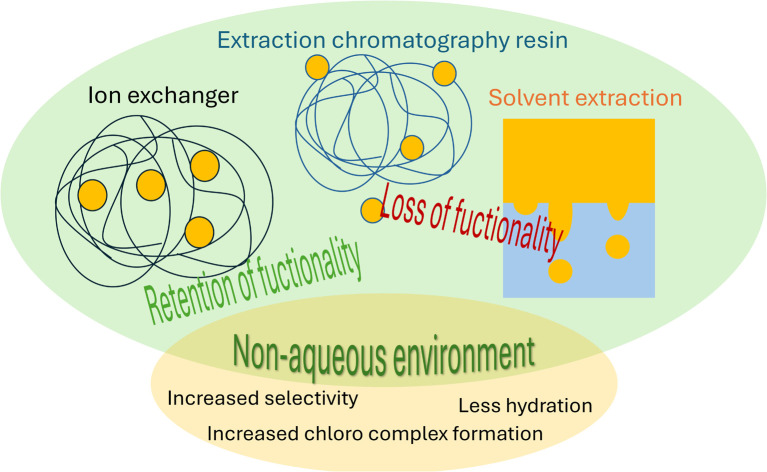

## 1. Introduction

High-purity rare-earth elements (REEs) are indispensable in many modern-day technologies, such as permanent magnets in electrical motors and wind turbines, catalysts for emission control, advanced ceramics and alloys, and lasers. The purification of the (individual) REEs, however, remains one of the most challenging tasks in hydrometallurgy up to this day. Before the introduction of industrial-scale solvent extraction, ion exchange was the main technique for rare-earth separation.^1,2^ Ion exchange is still often included as one of the main techniques for obtaining high-purity REEs on a smaller scale. Cation exchange combined with elution using complexing agents such as ethylenediaminetetraacetic acid (EDTA), or nitrilotriacetic acid, is the most frequently used form of ion exchange for REE purification industrially.^2^ While charge, size and degree of solvation of the exchanged ions influence the sorption onto a cation exchanger, the stability constants of the REE complexes with the eluting ligands are controlling the separation of each individual REE. In contrast, anion exchange has primarily been used for chromatographic separation of REEs as an analytical technique, yet quaternary ammonium functionalised exchangers proved to be interesting for REE purification from transition metal impurities.^3^ However, differences in the sorption of REEs are limited when extracting from acidic aqueous solutions, as the affinity of individual neighboring REEs for most ion exchangers is almost identical due to the limited differences in their ion radius. The addition of polar organic solvents often improves the selectivity of the ion exchange resins.^2–4^ Hubicki and Olszak showed enhanced separation of yttrium and neodymium from a nitrate solution (0.1–7 mol L^−1^) containing up to 95 vol% methanol and acetone.^5^ It was suggested that the selectivity for Nd(iii) over Y(iii) in nitrate medium resulted from the formation of anionic Nd(iii) species with a higher charge compared to the Y(iii) anionic species, *i.e.* Nd(NO_3_)_5_^2−^*versus* Y(NO_3_)_4_^−^.^6^ A follow-up study revealed that the addition of a co-solvent, *i.e.* acetic acid, tri-*n*-butyl phosphate, dimethyl sulfoxide (DMSO), formamide, *N*,*N*-dimethylformamide (DMF) and dioxane, to the methanol solution decreased the recovery and separation factor of the REEs.^7^ It was proposed that an increase in solvent donor number promoted solvation and decreased the formation of anionic complexes. Similar studies which involve the use of polar organic solvents in ion exchange have found that the highest distribution coefficients are found in systems using solvents with the lowest dielectric constants (methanol, ethanol).^8–10^ The increased sorption is caused by an increased tendency to form (anionic) complexes in solvents with low dielectric constants. It was furthermore suggested that differences in solvation, in kinetics or in the stability of the complexes found on the ion exchanger would explain the larger variations of the affinity of the REEs for the resin. Avdibegović *et al.* showed that the addition of ethanol greatly influences the efficiency and selectivity of the recovery of the transition metal ions Fe(ii), Co(ii), Ni(ii), Mn(ii) and Cu(ii) from sulfate solution, using the strong base anion exchanger Amberlite IRA-402 (chloride form).^3^ Both Cu(ii) and Fe(ii) were efficiently recovered from 80 vol% ethanolic solutions, while leaving the other metal ions in solution. Surprisingly, when increasing the ethanol concentration further to 95 vol%, Fe(ii) recovery decreased significantly, and Cu(ii) was selectively recovered.

Similarly, recent investigations into the (partial) replacement of the aqueous phase in solvent extraction by a polar molecular organic solvent (PMOS), *i.e.* non-aqueous solvent extraction, have shown increased extraction efficiency and enhanced separation of REEs from ethylene glycol or poly(ethylene) glycol 200 (PEG-200) solution by the neutral extractant Cyanex 923.^11–14^ The combination of increased ion-pair formation and reduced solvation strength both were suggested to increase extraction efficiency of rare-earth chlorides from various PMOSs.^15^ A drawback of solvent extraction, and non-aqueous solvent extraction in particular, is the mutual solubility of the two phases, leading to losses of extractant or other organic components.^16^ This not only causes economic loss, but an environmental challenge as well. While organic compounds can be removed from aqueous solutions by, for instance, activated carbon, this approach is not possible from PMOSs used in non-aqueous solvent extraction.

Present paper studies the effects of PMOSs on the sorption of rare-earth (La, Nd, Eu, Dy, Yb) and (divalent) transition metal (Fe, Co, Cu, Ni, Mn) chloro complexes by a strong anion exchanger resin, Amberlite IRA-402 (chloride form), and extraction chromatography resins, TEVA (chloride form) and DGA resin. TEVA (chloride form) resin incorporates the quaternary ammonium extractant methyltrioctylammonium chloride (Aliquat 336) as the active ion exchange functionality, and DGA resin contains the chelating agent *N*,*N*,*N*′,*N*′-tetraoctyl diglycolamide (TODGA). The results obtained with the ion exchanger or extraction chromatography resins are compared with non-aqueous solvent extraction using Aliquat 336 and TODGA, which are the active components in TEVA and DGA resin, respectively. Based on this, current paper will address the applicability of non-aqueous solvents in combination with anion exchange resins and extraction chromatography resins, specifically focusing on the loss of extractants or functionality to the PMOSs, and how it compares to the mutual solubility observed in non-aqueous solvent extraction.

## 2. Experimental

### 2.1. Materials

Ethanol (absolute, 99.8%), formamide (99.5%), hydrochloric acid (density 1.18 g mL^−1^, ∼37 wt%), anhydrous CuCl_2_ (99%), MnCl_2_·4H_2_O (>99%), and Aliquat 336 (mixture of quaternary ammonium compounds, tri-C_8-10_-alkylmethyl chlorides) were purchased from Thermo Fisher Scientific (Geel, Belgium). NiCl_2_·6H_2_O (>97%), CoCl_2_·6H_2_O (>98%), and the ICP standards of Fe, Co, Cu, Ni, Mn, La, Nd, Eu, Dy, Yb and Sc (1000 mg L^−1^, in 2–5 wt% HNO_3_) were obtained from Chem-Lab nv (Zedelgem, Belgium). FeCl_2_ (98%) and ethylene glycol (>99%) were purchased from Merck (Darmstadt, Germany). Amberlite^®^ IRA 402 chloride form (gel-type, styrene-divinylbenzene with quaternary ammonium functional group, and particle size 600–750 μm) was obtained from Fluka Analytics (Buchs, Switzerland). TEVA resin (strong anion exchanger with trioctylmethylammonium chloride as active component, particle size 50–100 μm) and DGA normal resin (chromatographic resin with *N*,*N*,*N*′,*N*′-tetra-*n*-octyldiglycolamide as active component, particle size 50–100 μm) were obtained from Eichrom (Lisle, Illinois, USA) *via* Triskem (Bruz, France). *N*,*N*,*N*′,*N*′-Tetraoctyl diglycolamide (TODGA, 95%) was obtained from Amadis Chemical (Hangzhou, China). Shellsol A150 (aromatic diluent) and Shellsol GS190 (aliphatic diluent) were provided by Shell Global Solutions (Amsterdam, The Netherlands). Water was always of ultrapure quality (<0.055 μS cm^−1^ at 298.15 K) using a Merck Millipore Milli-Q Reference A+ system.

### 2.2. Instrumentation

The elemental analysis of the aqueous and mixed aqueous-organic solutions was determined by Inductively Coupled Plasma Optical Emission Spectroscopy (ICP-OES). The spectrometer (Avio 500 Perkin-Elmer, USA) was equipped with a GemCone High Solids nebulizer, baffled cyclonic spray chamber, 2.0 mm inner diameter alumina injector and PerkinElmer Hybrid XLT torch. All samples (including quality controls and calibration standards) were diluted using a 2 wt% HNO_3_ solution to reach the desired concentration range, and measured in triplicate. Scandium (5 mg L^−1^) was used as internal standard. The calibration curves were constructed by measurement of standard solutions spanning the expected sample concentration range. Particle size measurements were performed using a Mastersizer Hydro SV laser diffractometer (Malvern Panalytical, UK). Obscuration was limited between 3% and 10%. Each sample was measured in quintuplicate in volume distribution mode. Moisture analysis of the resins was performed by a HC103 moisture analyzer (Mettler-Toledo, USA). Approximately 1 g of material was treated with a heat-and-hold program at 150 °C, with the stop condition being a Δwt% < 1 mg in 50 seconds. A Shimadzu LCMS-2020 system using a DUIS-2020 dual ion source in ESI + APCI +/- mode was used to investigate the loss of Aliquat 336 or TODGA from TEVA and DGA resins, respectively, in aqueous, ethanolic, ethylene glycol and formamide solutions. The column used was a InfinityLab Poroshell 120 EC-C18 2.7 μm 2.1 × 100 mm. Calibration curves were made for TODGA (based on PDA chromatogram at 215 nm) and for the four main compounds that make up Aliquat 336: trioctylmethylammonium (*m*/*z* 368), dioctyldecylmethylammonium (*m*/*z* 396), octyldidecylmethylammonium (*m*/*z* 424) and tridecylmethylammonium (*m*/*z* 452). Depending on the expected Aliquat 336 or TODGA concentration in the solutions, the samples were not diluted (DGA + water, ethylene glycol, formamide), 10 times diluted (DGA + ethanol) or 100 times diluted (TEVA samples) using methanol. An injection volume of 1 μL was used. The composition of eluent was varied from 50 vol% methanol in water (+0.1% formic acid) to 100 vol% methanol (+0.1% formic acid) over a time range of 5 minutes, followed by 10 minutes of hold.

### 2.3. Batch ion exchange and solvent extraction experiments

The feed solutions containing Fe(ii), Cu(ii), Co(ii), Ni(ii), Mn(ii) and the lanthanides representative for the REEs series, *i.e.* La(iii), Nd(iii), Eu(iii), Dy(iii) and Yb(iii) were prepared from their (hydrated) chloride salts, by dissolving these in water to the desired concentration (50 mmol L^−1^ or 200 mmol L^−1^). These concentrated feeds were further diluted with water or organic solvents (*i.e.* 50, 80 or 95 vol% ethanol, ethylene glycol or formamide), and HCl was added (0.01–0.2 mol L^−1^). The concentration in this multi-element feed was 0.5 mmol L^−1^ per element, unless stated otherwise. Note that Fe(ii) has the strong tendency to be largely oxidised to Fe(iii) in concentrated ethanolic solution, as mentioned by Avdibegović *et al.*^3^ This must be taken into account when interpreting the results. In contrast, the rate of Fe(ii) oxidation in ethylene glycol and formamide solutions is much slower than in ethanolic solutions, which makes interpretation less troublesome.^17^ Hence, feed solutions were always freshly prepared before each experiment. A volume of 2.5 mL of the diluted feed solutions were brought in contact with 0.025 g of resin in 4 mL glass vials, and shaken at 300 rpm using an orbital shaker for 60 minutes at room temperature, unless stated otherwise. The tests have been performed without taking into account the moisture content of the resins, which is 49% for Amberlite IRA-402, 4% for TEVA and 1% for DGA resin. Experiments with dried resins, however, did not yield any significantly different results. The resin was subsequently separated from the solution by filtration using syringe filters (0.45 μm pore size). The metal concentrations in the feed before contact with the resin and the recovered solutions after ion exchange were measured by ICP-OES (*vide supra*). The recovery percentage of the metal by the resin was calculated using eqn (1), with *c*_i_ and *c*_f_ the initial metal feed concentration and the metal concentration after contact with the resin, respectively. The distribution coefficient *K*_D_ is expressed by eqn (2), in which *V* is the volume (L) of the solution and *m* is the mass (g) of the resin added. The separation factor between two metal ions A and B, *α*_A,B_, is described by eqn (3), where *α*_A,B_ ≥ 1.1
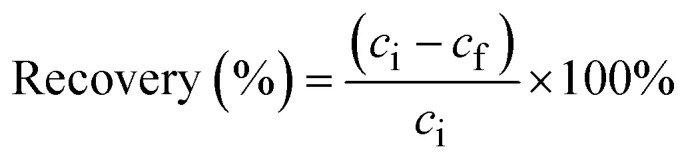
2
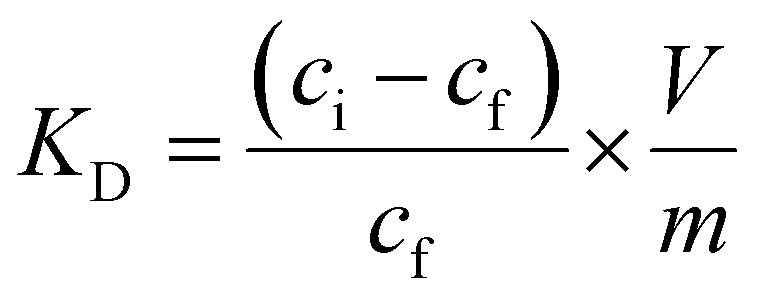
3
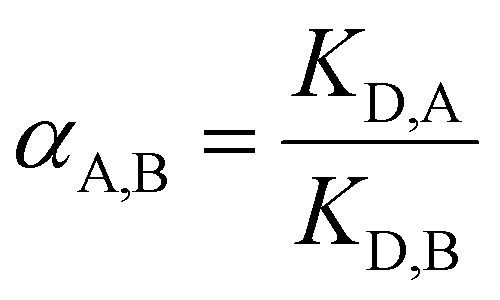


Feed solutions, consisting of 50 vol% ethanol, 95 vol% ethylene glycol or 95 vol% formamide and containing 0.2 mol per L HCl and 2 mmol L^−1^ of each element, were used in batch solvent extraction experiments. 4 mL of the feed solution was contacted with 4 mL of an organic phase containing 20, 40 or 60 vol% of either Aliquat 336 or TODGA, diluted in an aromatic diluent (Shellsol A150) or an aliphatic diluent (Shellsol GS190). The experiment was caried out in graduated 15 mL centrifuge tubes, which were shaken by a FinePCR Ash 3 Arm shaker for 30 minutes at room temperature, after which the samples were centrifuged. The aqueous, ethanolic, ethylene glycol or formamide phases were subsequently sampled and the elemental concentrations were measured by ICP-OES (*vide supra*). The calculation of the percentage extraction (%*E*) is identical to the expression for the recovery percentage in eqn (1), with *c*_i_ and *c*_f_ the initial metal feed concentration and the metal concentration in the raffinate after solvent extraction, respectively.

## 3. Results and discussion

A strong-base anion exchanger Amberlite IRA-402 (chloride form), and two extraction chromatography resins, *i.e.* TEVA (chloride form) resin, containing quaternary ammonium functionalities, and a diglycolamide (DGA) resin, containing the tridentate ligand *N*,*N*,*N*′,*N*′-tetra-*n*-octyl diglycolamide (TODGA), were tested in batch experiments using feed solutions containing varying concentrations of a polar molecular organic solvent (PMOS). The PMOSs have a large impact on the ion exchange reaction, as the solvent dielectric constant and the donor properties of the solvent influence metal ion complex formation, and hence impact the metal ion recovery by the resin. This can be illustrated by the extraction mechanism for the ammonium-based and glycolamide-based resins and extractants, shown in eqn (4) and (5), respectively, with the overbar signifying species in the resin (IX) or organic phase (SX).^18,19^ Lower dielectric constants would increase the formation of the anionic or neutral metal complexes in PMOSs, and hence extraction would be increased. However, these complexes are of course solvated, so that, if the solvent in question would coordinate strongly with the metal ions or metal complexes (*e.g.* high Gutmann donor number), the extraction would instead be hindered.^15^4
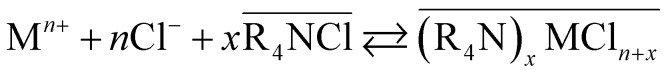
5
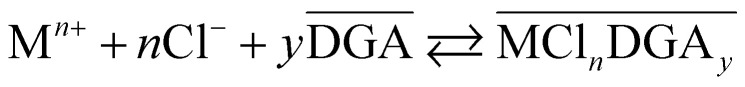


The following sub-sections will discuss the effect of PMOSs (ethanol, ethylene glycol and formamide) on the separation of metal ions by various resins, as well as their effect on solvent extraction using extractants identical to functionalities in the studied resins.

### 3.1. Effect of PMOSs on metal uptake by ion exchange resin

**Fig. 1:**
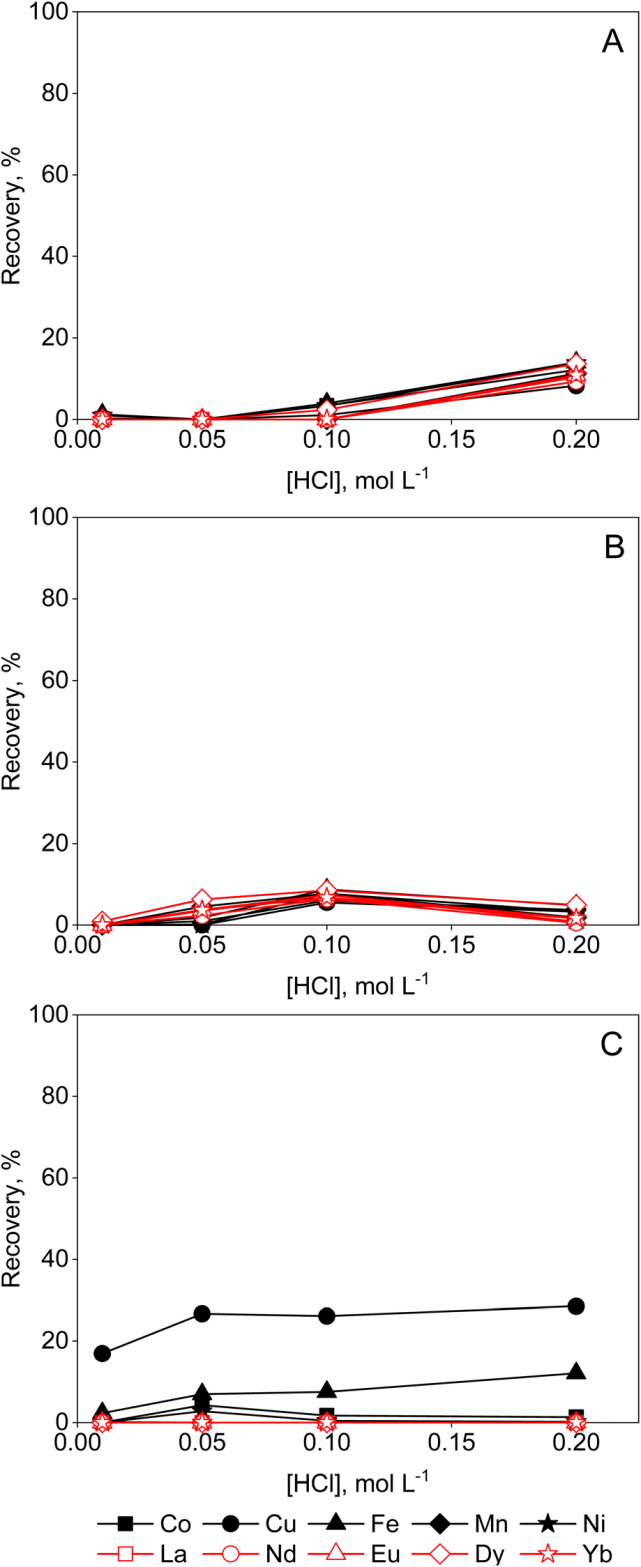
Effect of HCl concentration on the sorption of transition metals and REEs from feeds containing (A) 50, (B) 80 or (C) 95 vol% ethylene glycol by Amberlite IRA 402 (chloride form). Conditions: 25 mg of resin, 2.5 mL of feed solution, metal concentration 0.5 mmol L^−1^ (each), *t* = 30 min, *T* = 21 ± 1 °C.

**Fig. 2:**
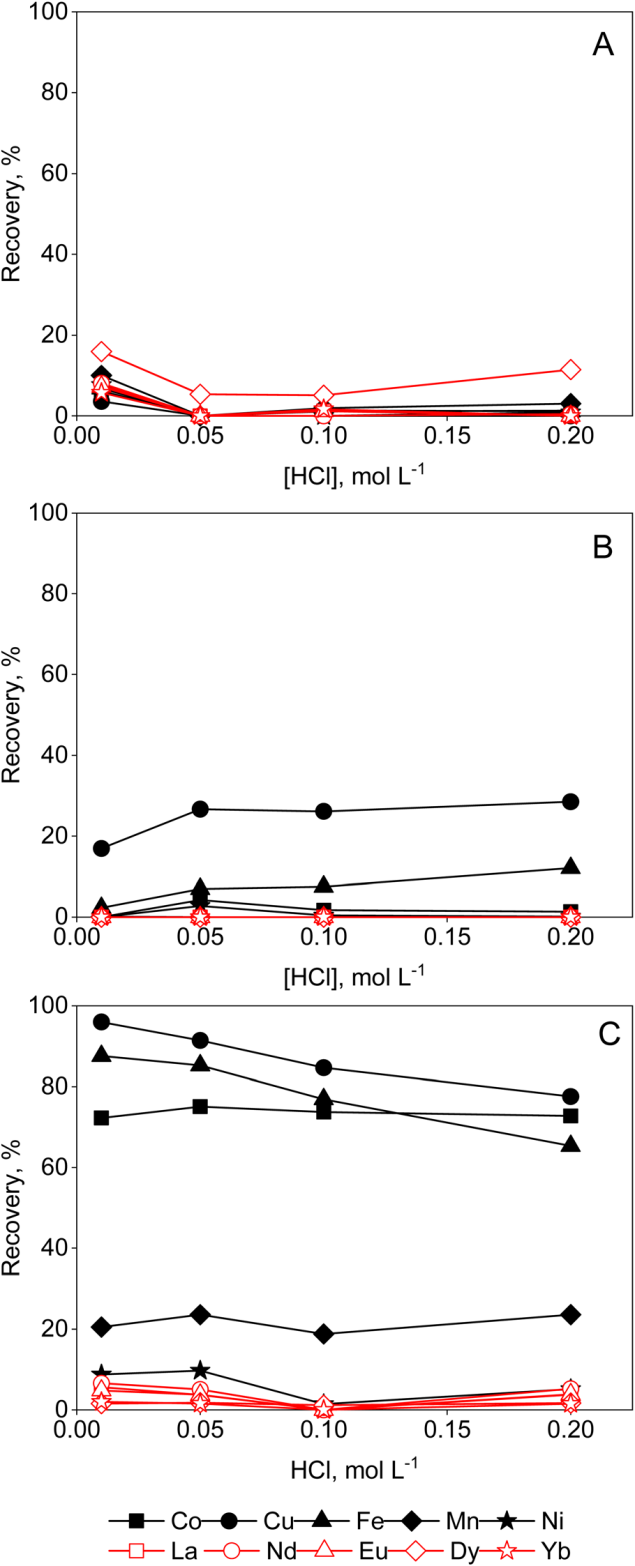
Effect of HCl concentration on the sorption of transition metals and REEs from feeds containing (A) 50, (B) 80 or (C) 95 vol% ethanol by Amberlite IRA 402 (chloride form). Conditions: 25 mg of resin, 2.5 mL of feed solution, metal concentration 0.5 mmol L^−1^ (each), *t* = 30 min, *T* = 21 ± 1 °C.

**Fig. 3:**
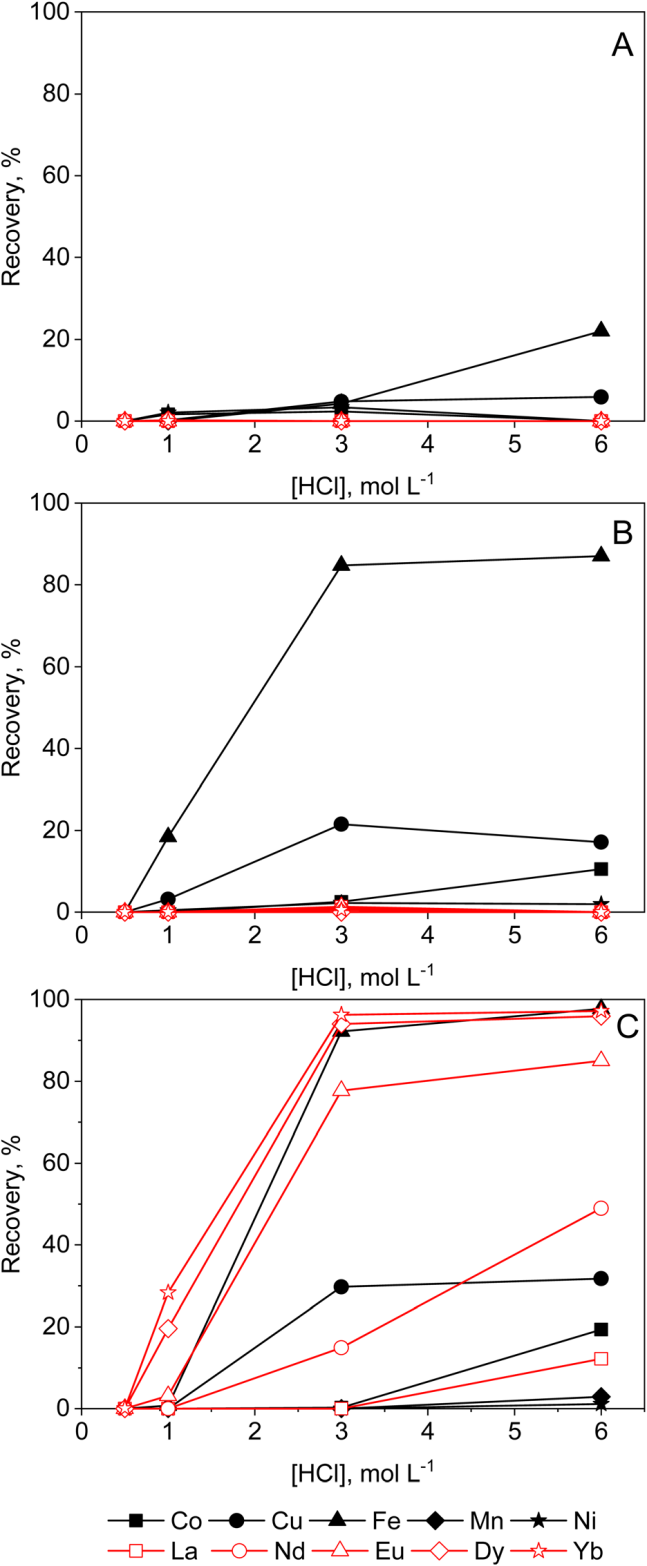
Effect of HCl (0.5–6 mol L^−1^) on the sorption of transition metals and REEs from aqueous feeds by (A) Amberlite IRA 402 (chloride form), (B) TEVA (chloride form) and (C) DGA resin. Conditions: 25 mg of resin, 2.5 mL of feed solution, metal concentration 0.5 mmol L^−1^ (each), *t* = 30 min, *T* = 21 ± 1 °C.

Fig. 1 shows that there is a slight increase in the recovery of metal complexes on Amberlite IRA 402 resin when increasing the ethylene glycol concentration to 95 vol%, particularly for Cu(ii) and Fe(ii). This effect is more pronounced in ethanolic compared to ethylene glycol solutions (Fig. 2). In contrast, in aqueous and formamide solutions, recoveries are low (Fig. S1 and 2†). This correlates with the static dielectric constants *ε* (at 25 °C), which are given in Table 1, and which increase when water is added to ethanol or ethylene glycol, while addition of water decreases the dielectric constant of formamide (see the ESI† for the calculation of the dielectric constants of mixtures). The formation of anionic metal complexes is enhanced in solvents with a lower dielectric constant. While addition of a PMOS with a lower dielectric constant thus significantly enhances the recovery of transition metals by the Amberlite IRA 402 resin, the increase in HCl concentration up to 6 mol L^−1^ in aqueous solutions has only limited effect (Fig. 3A). In the case of ethylene glycol or ethanol feed solution, the effect of HCl concentration, with an upper limit of 0.2 mol L^−1^ due to constraints on the water content, is rather small. The REEs were not recovered efficiently by the anion exchanger, as they are assumed not to form anionic complexes with chloride ions due to strong hydration, even at 95 vol% of PMOS.^20^ Cu(ii), Co(ii) and Fe(ii)/Fe(iii), on the other hand, tend to form anionic complexes at higher chloride concentration or in solvents with a lower dielectric constant, usually in the form of [MCl_4_]^2−^ (M = Cu(ii), Co(ii) or Fe(ii)) or [FeCl_4_]^−^ in the case of Fe(iii).^21^ Ni(ii) does not form tetrahedral chloro complexes, since it prefers to maintain the octahedral hexaaqua complex, explaining the negligible uptake by the anion exchanger.^22^ Efficient removal of Fe(ii), Cu(ii) and Co(ii), which are important transition metal impurities in NdFeB and SmCo permanent magnets, can thus be performed using feed solutions containing 95 vol% EtOH and 0.01 mol per L HCl. The separation factors *α*_Cu,Nd_, *α*_Fe,Nd_, and *α*_Co,Nd_ reached up to 337, 99, 37, respectively, in this particular system.

**Table 1:** Dielectric constants *ε* calculated in OLI Studio V11.5 using the mixed-solvent electrolyte framework (OLI Systems Inc., Parsippany NJ) for the solvent mixtures used in present paper, temperature 25 °C

Vol% solvent	Formamide	Ethylene glycol	Ethanol
0 (water)	78.3	78.3	78.3
50	93.9	68.7	51.5
80	103	56.0	35.3
95	108	44.2	27.1

### 3.2. Effect of PMOSs on metal uptake in extraction chromatography resins

**Fig. 4:**
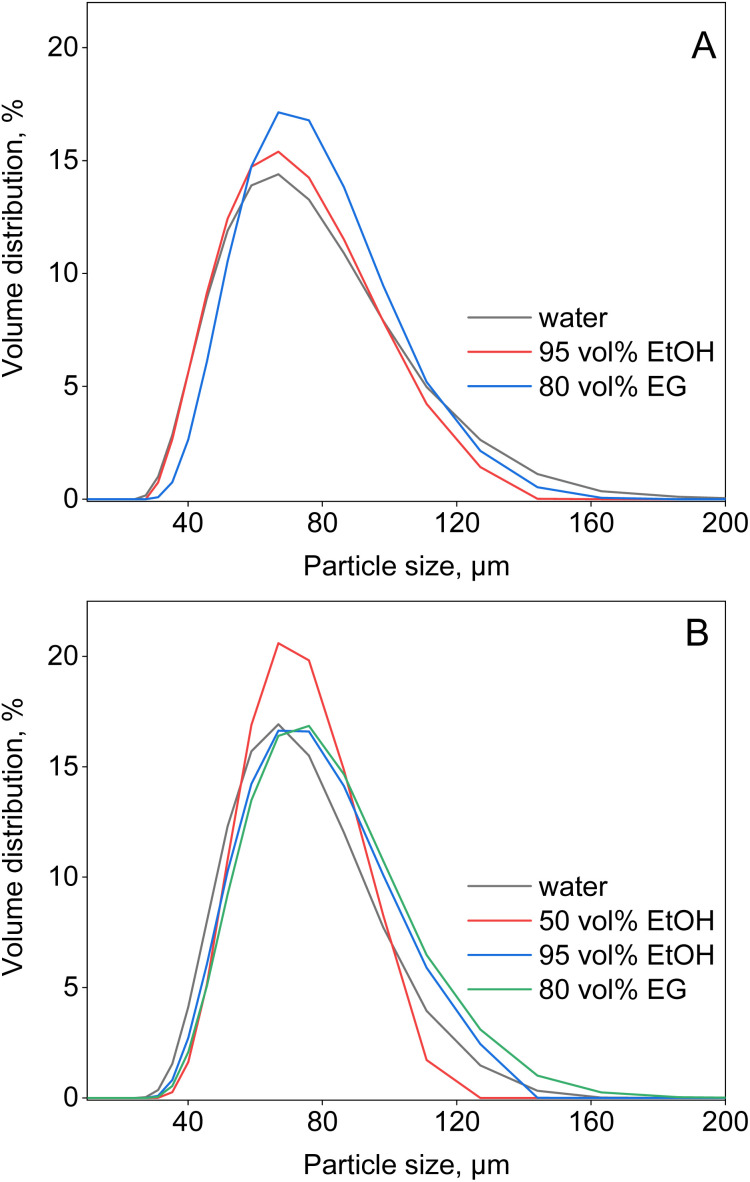
Effect of solvents on the particle size of (A) TEVA (chloride form) and (B) DGA resin at room temperature.

Batch tests similar to those performed with Amberlite IRA 402 resin have been performed with TEVA resin. TEVA is a commercially available strong anion exchanger, with the active component Aliquat 336 incorporated in the cross-linked polymeric matrix. In solvent extraction, the extractant Aliquat 336 can efficiently separate REEs from transition metals from ethylene glycol (+LiCl) solutions.^16^ One of the drawbacks of non-aqueous solvent extraction, however, is the mutual solubility of the so-called more-polar (MP) phase and less-polar (LP) phase (*vide infra*). Hence, it would make sense to screen the TEVA resin, since the Aliquat 336 molecules should be confined within the polymeric matrix, avoiding the loss of extractant. Nonetheless, the results shown in Fig. S3 (see ESI†) indicate that there is only limited uptake of metal ions by TEVA resin in any of the tested feed solutions, in contrast to the results for the Amberlite resin, which has chemically similar functionalities. While slower kinetics might have been a reason for this difference, this was not observed during the kinetics studies (ESI, Fig. S4†). This is supported by studying the effect of ethanol and ethylene glycol on the swelling of the TEVA resin, studied by laser diffraction, as less polar solvents might reduce the swelling and slow down ion exchange (Fig. 4A).^4^ However, no significant differences compared to water were observed. Hence, the loss of Aliquat 336 from TEVA resin in the PMOSs and water might explain the poor performance of TEVA resin. About 100 mg (±2 mg) of resin was contacted with 10 mL of each PMOS or water for 1 hour. The solvents were then filtered, and quantitatively analysed using LC-MS. Since Aliquat 336 is a mixture of compounds, concentrations of the four main compounds have been analyzed: trioctylmethylammonium (TOMA), dioctyldecylmethylammonium (DODMA), octyldidecylmethylammonium (ODDMA) and tridecylmethylammonium (TDMA). Indeed, Table 2 shows that the presence of Aliquat 336 was confirmed in all PMOSs, and even in water. The concentration of the least polar Aliquat 366 compounds, ODDMA and TDMA, was negligible in water. Hence, due to the loss of extractant, it is not recommended to use TEVA resin in combination with PMOSs. Even more surprising was the apparent loss of Aliquat 336 to pure water, yet more in depth investigations would be needed to determine the extent of the loss of functionality in water at various conditions. These results are in contrast with the Amberlite IRA-402 anion exchanger, which has immobilised quaternary ammonium functionalities, and hence does not experience loss of retention capacity in PMOSs.

**Table 2:** Concentrations of TODGA from DGA resin and Aliquat 336 from TEVA resin found in different solvent systems. Experimental conditions: 100 mg resin contacted for 1 h with 10 mL of solvent at room temperature

Solvent system	[TOGDA]_loss_ DGA resin, mg L^−1^	[Aliquat 336]_loss_ TEVA resin, mg L^−1^			
		TOMA	DODMA	ODDMA	TDMA
Water	3.00	703.10	206.71	10.12	0.00
95 vol% ethanol	4328.52	995.08	1376.79	1058.48	266.88
95 vol% ethylene glycol	20.74	1065.42	1381.84	1008.28	225.25
95 vol% formamide	23.04	1113.93	1414.40	1079.13	241.85

**Fig. 5:**
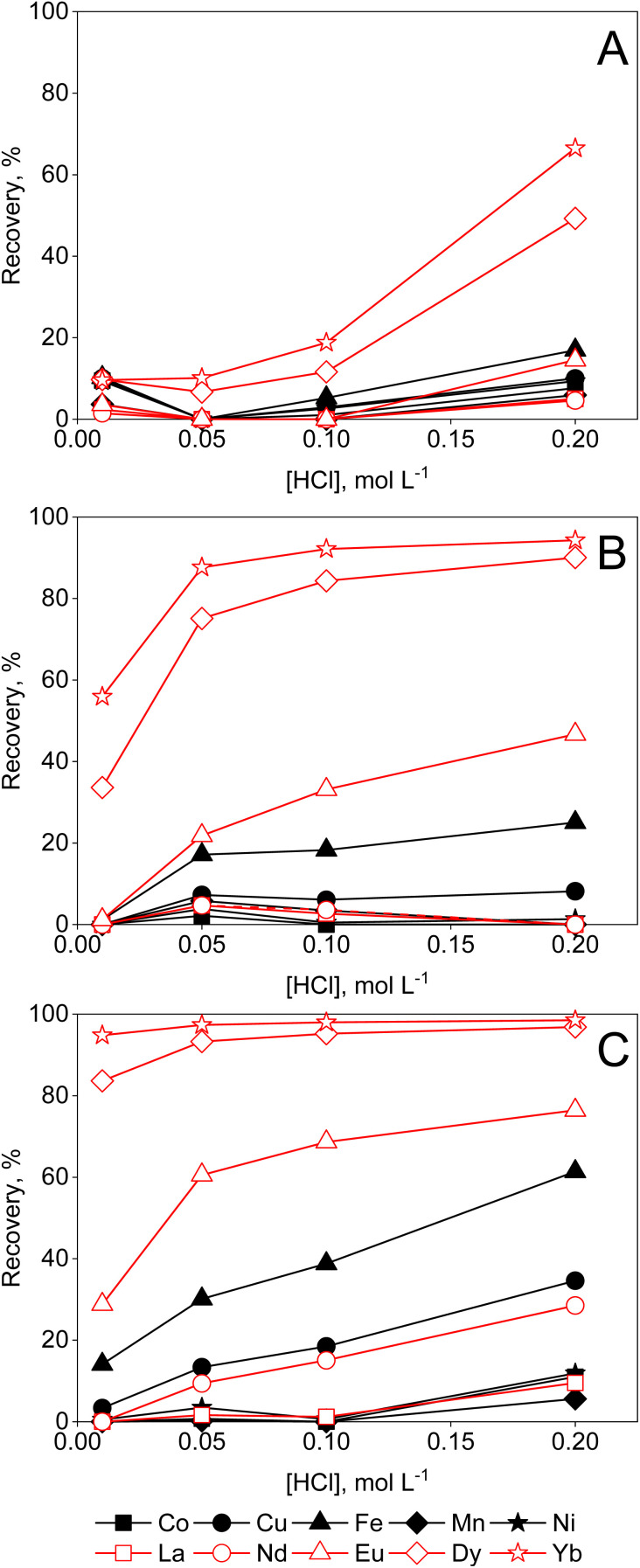
Effect of HCl concentration on the sorption of transition metals and REEs from feeds containing (A) 50, (B) 80 or (C) 95 vol% ethylene glycol by DGA resin. Conditions: 25 mg of resin, 2.5 mL of feed solution, metal concentration 0.5 mmol L^−1^ (each), *t* = 30 min, *T* = 21 ± 1 °C.

**Fig. 6:**
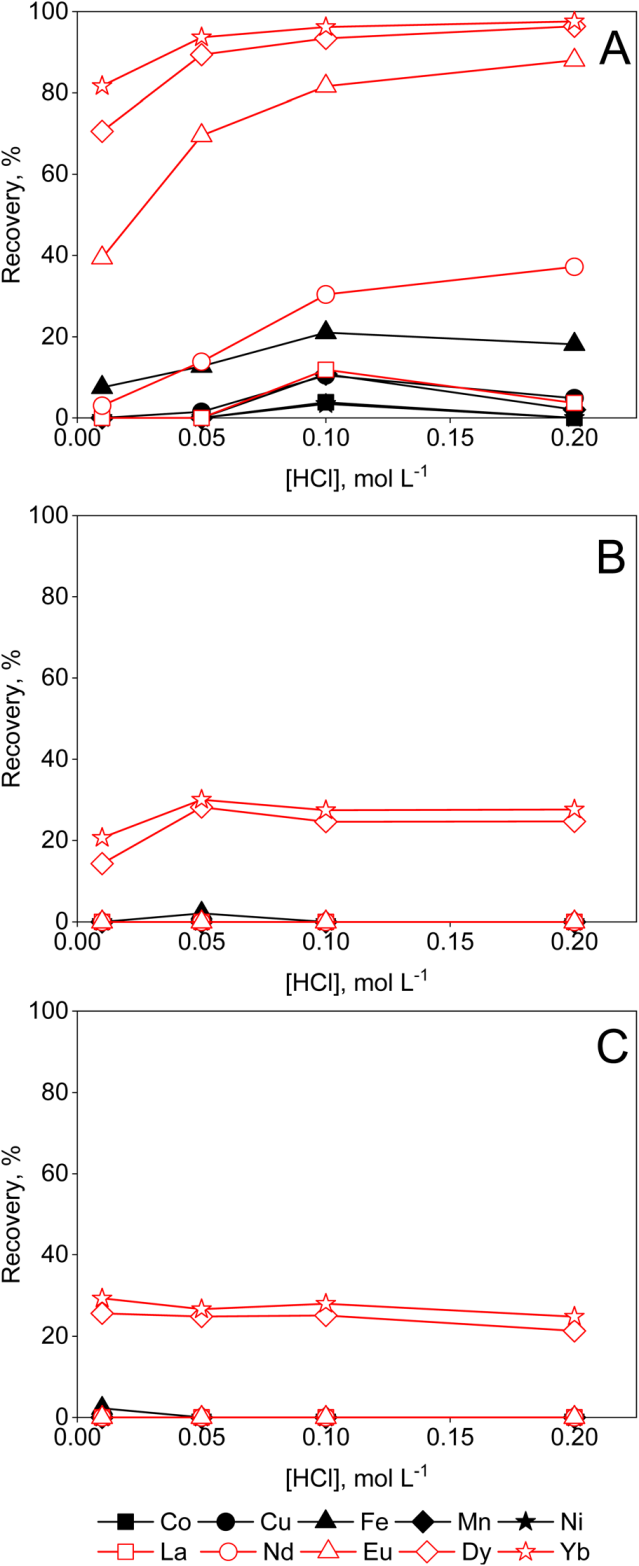
Effect of HCl concentration on the sorption of transition metals and REEs from feeds containing (A) 50, (B) 80 or (C) 95 vol% ethanol by DGA resin. Conditions: 25 mg of resin, 2.5 mL of feed solution, metal concentration 0.5 mmol L^−1^ (each), *t* = 30 min, *T* = 21 ± 1 °C.

DGA ligands are known for their high capacity and selectivity for REEs, and relatively low affinity for competing impurities. From aqueous solutions, they exhibit high distribution ratios at high acid concentrations. The extraction data in Fig. 3, show that there is efficient extraction of heavy REEs from aqueous solutions with HCl concentrations of 3 mol per L HCl or higher, which is in agreement with the literature.^18^ The recovery efficiencies from lean REE solutions are limited. Previous studies have shown how the REE recovery from diluted REE solutions can be improved, for instance by synthesis of novel DGA-based ligands.^23^ However, this is not compatible with the ‘Twelve Principles of Circular Hydrometallurgy’, *i.e.* chemical diversity should be reduced if possible.^24^ While high acid concentrations improve the uptake of the REEs by DGA resin, a similar effect can be obtained by the addition of a PMOS to aqueous solutions instead (Fig. 5 and 6) avoiding the need for novel extractants. A steady increase in recovery yields of REEs can be observed with increasing ethylene glycol concentration in the feed (Fig. 5). Transition metals were not taken up by the resin, with the exception of Fe(ii) and Cu(ii), which were sorbed by the resin only at higher ethylene glycol concentrations. Furthermore, a distinct increase in REE recovery was obtained by increasing the HCl concentration to 0.2 mol L^−1^. This is still much lower than the acid concentrations required in aqueous solutions for efficient REE recovery by DGA, hence reducing corrosivity and costs. The use of ethylene glycol might be of interest for the intra-REE separation using DGA resins, as heavy REEs (HREEs) seem to be preferentially recovered over the light REEs (LREEs). From solutions containing 95 vol% ethylene glycol and 0.01 mol per L HCl, no detectable amount of La(iii) or Nd(iii) was sorbed, and only Eu(iii), Dy(iii) and Yb(iii) were recovered on the resin. As to the kinetics of the sorption (ESI, Fig. S5B†), a clear increase in iron removal can be seen from ethylene glycol solutions, though the recovery percentage Cu(ii) decreased. Since is Fe(ii) slowly oxidised over time in organic solvents (*vide supra*), and this reaction can be catalysed by the reduction of Cu(ii),^25^ an exchange of the harder Lewis acid Fe(iii), replacing the soft Lewis acid Cu(i) might take place, as the latter is less strongly sorbed on the resin. For the recovery from ethanolic solutions, the results are somewhat unexpected, since an increase in ethanol concentration beyond 50 vol% leads to a decrease of the uptake of REEs (Fig. 6). The swelling of the resin was investigated, as shrinkage of resin particles due to the low polarity of ethanol could explain the reduced availability of the available sorption sites, causing the observed sudden decrease in sorption. However, no large changes in terms of particle size were observed in 50 vol% or 95 vol% ethanol compared to water (Fig. 4B). It is possible that part of the DGA ligand, which is not physically bound to the resin, is leached out when contacted with higher concentrations of ethanol, thus reducing the capacity of the resin. This might be further corroborated by the decrease of uptake of metal ions over a 168-hour period (ESI, Fig. S5†). Mass spectrometry investigations on a 95 vol% ethanolic solution contacted with DGA resin for 1 hour confirmed the presence of TODGA in ethanolic solutions (Table 2), further proving that the lower metal uptake by the resin is caused by the extractant being leached out. Hence, the use of ethanol in combination with the DGA resin is not recommended. Similar analysis in ethylene glycol, formamide and aqueous solution showed only limited loss of TODGA from the resin. The recovery yields of metal ions from aqueous and formamide solutions were poor in any of the studied conditions (ESI, Fig. S6 and S7†), since ion-pair formation is very limited in solvents with high dielectric constants.

### 3.3. Solvent extraction tests with TODGA and Aliquat 336

Solvent extraction is the distribution of metal ions between two liquid phases, a more polar (MP) feed phase and a less polar (LP) phase containing an extractant, diluent and sometimes a modifier or a synergist. In contrast, in ion exchange or extraction chromatography the extraction medium is a solid phase, having a limited number of (charged) functional groups which can retain metal complexes. As the number of these functional groups per gram of resin material is limited, the capacity for retention of elements is limited as well, restricting the use of ion exchange to dilute feeds with concentrations in the ppm range.^24^ In contrast, industrial solvent extraction processes uses feeds up to hundreds of grams per litre of metal ions. However, solvent extraction is rarely used in case of dilute feed and waste solutions, as this requires high aqueous-to-organic ratios, thus leading to high losses of the organic to the aqueous phase, causing contamination issues and financial costs. Hence, for dilute feeds, ion exchange and extraction chromatography are the most suitable recovery techniques.^24^ Moreover, when non-aqueous solvent extraction is considered, also increased mutual solubility of the LP phase into the MP phase, and *vice versa*, has to be considered.^26,27^ As the functional groups of ion exchangers and chromatographic resins are either covalently bound to a resin framework, or at least contained within the resin, little to no losses of functionality are to be expected. The results described in previous subsection, however, indicate that the latter statement is not always true for the chromatographic resins.

Hence, it is evident that solvent extraction and ion exchange or extraction chromatography each have their specific application based on the concentration of the feed, the end-product quality envisioned, and choices should be made based on economic and technical considerations. Since this paper specifically investigates feed solutions containing PMOSs, it is useful to highlight the possible advantages of non-aqueous ion exchange or extraction chromatography compared to non-aqueous solvent extraction, and to compare the capabilities of each technique for the recovery and separation of transition metals and REEs. In order to compare the advantages and disadvantages of both non-aqueous ion exchange and non-aqueous extraction chromatography to non-aqueous solvent extraction, solvent extraction tests have been performed with similar feeds having metal concentrations of 2.0 mmol L^−1^ for each element (20 mmol L^−1^ total) and 0.2 mol per L HCl in ethanol (50 or 95 vol%), ethylene glycol (95 vol%) and formamide (95 vol%). These feeds were contacted with 20, 40 or 60 vol% of Aliquat 336 (A336, diluted in Shellsol A150) or TODGA (diluted in Shellsol A150 or GS190). A clear volume change can be visually observed in all tested conditions, most strikingly in the samples with Aliquat 336 as extractant. This observation confirms earlier literature reports regarding the solubility of Aliquat 336 in PMOSs.^16^ Also TODGA in combination with 50 vol% ethanol solutions showed volume changes at equilibrium. An overview of the phase ratios at equilibrium for all studied systems can be found in Table S1 (ESI).† While the extent of the mutual solubility of each component in the system was not quantified, it is evident that such behaviour is undesirable. The loss of extractant to the MP phase would put a lot of strain on the economic viability of a non-aqueous solvent extraction process. This contrasts with non-aqueous ion exchange and non-aqueous ion chromatography, where the functionalities are either chemically bound to an insoluble resin matrix or physically restrained by the resin matrix from dissolving into the PMOS in most conditions. However, careful consideration of the PMOS is necessary in combination with chromatographic resins, as non-chemically bound functionalities might still dissolve in some polar solvents (*vide supra*).

**Fig. 7:**
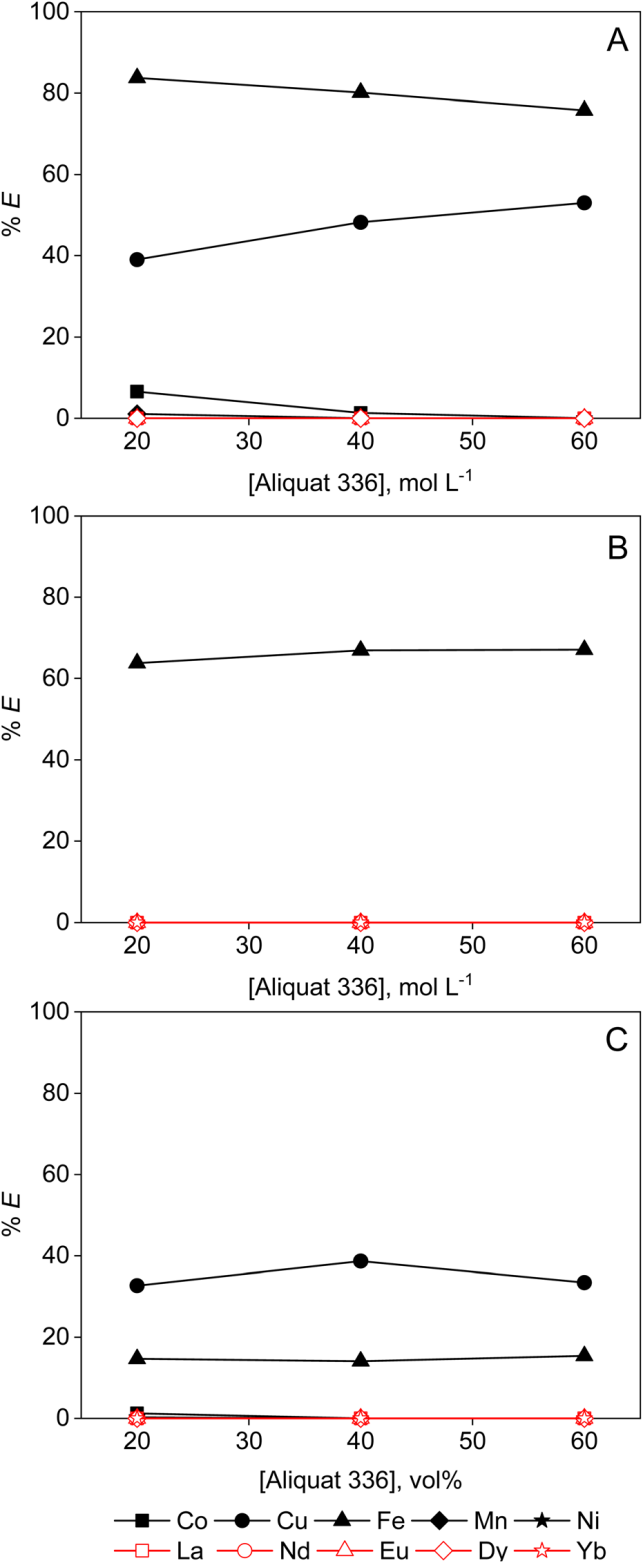
Extraction of transition metals and REEs from (A) 95 vol% ethylene glycol, (B) 50 vol% ethanol and (C) 95 vol% formamide by Aliquat 336 diluted in Shellsol A150. Conditions: [metal] = 2.0 mmol L^−1^ each, 0.2 mol per L HCl, LP : MP = 1 : 1 (4 mL : 4 mL), 15 min of contact time at room temperature.

The extraction efficiency of REEs by Aliquat 336 from any feed solution studied is negligible (Fig. 7). The extraction tests using 95 vol% ethanol are left out, as MP and LP phases were completely miscible in this case. Therefore, only the results for the experiments using 50 vol% ethanolic feeds are shown. Mainly Fe(ii) and Cu(ii) were extracted, with the extraction efficiencies for Fe(ii) increasing with decreasing dielectric constant of the MP solvent (ESI, Table S1†), *i.e.* about 40%, 60% and 80% extraction from formamide (95 vol%), ethanol (50 vol%) and ethylene glycol (95 vol%), respectively. Cu(ii) was not extracted at all from an ethanolic solution, which is probably due to the higher water concentration, *i.e.* 50 vol% water in the case of ethanol compared to only 5 vol% for the ethylene glycol and formamide extraction experiments. Hence, this reduces the activity of the chloride anion, and increases the solvation enthalpy of the extractable anionic complex.^28^ Compared with the TEVA resin, the solvent extraction process with Aliquat 336 did not perform better or worse, as in both cases losses of the extractant were observed. Comparing the solvent extraction results with those of the Amberlite IRA-402 anion exchanger, which also contains a quaternary ammonium functionality, a similar selectivity and recovery for Fe(ii) and Cu(ii) are observed. However, Amberlite IRA-402 resin has covalently bonded functional groups. Hence, non-aqueous ion exchange has a clear advantage over non-aqueous solvent extraction in this specific case.

**Fig. 8:**
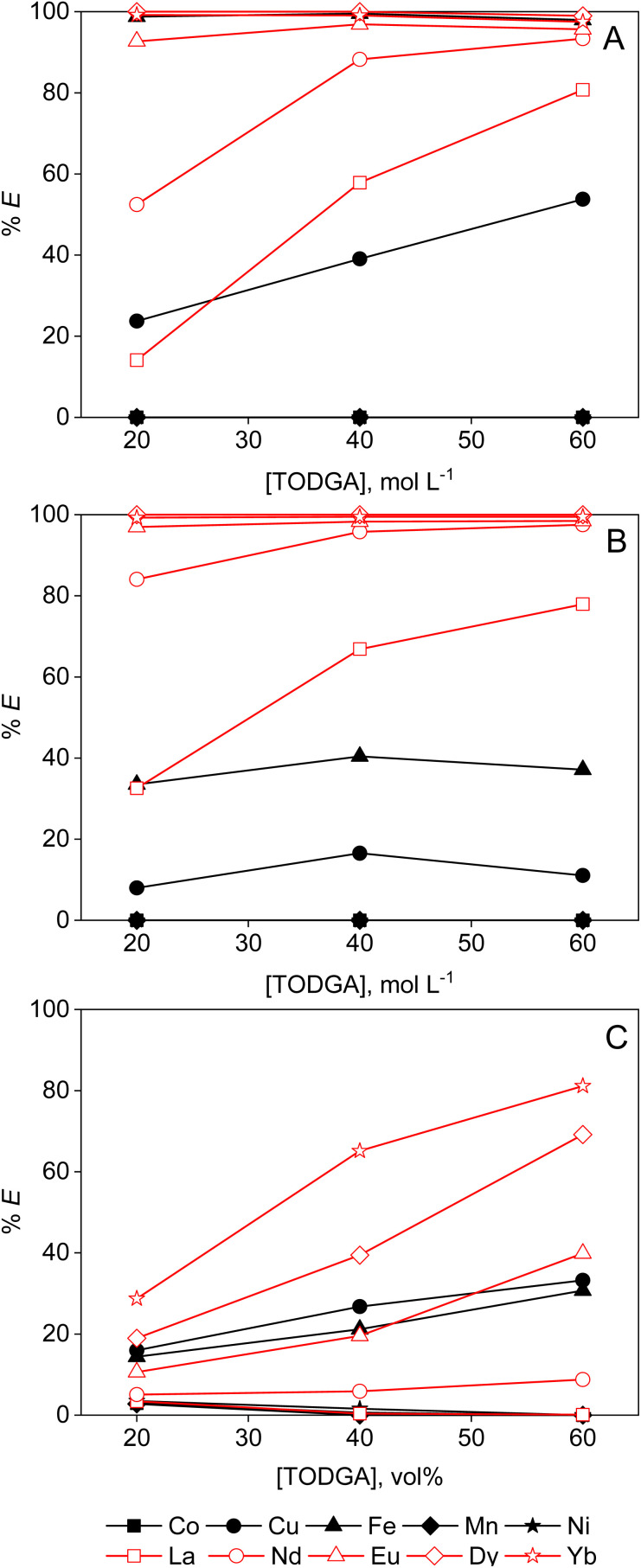
Extraction of transition metals and REEs from (A) 95 vol% ethylene glycol, (B) 50 vol% ethanol and (C) 95 vol% formamide by TODGA diluted in Shellsol GS190. Conditions: [metal] = 2.0 mmol L^−1^ each, 0.2 mol per L HCl, LP : MP = 1 : 1 (4 mL : 4 mL), 15 min of contact time at room temperature.

TODGA extracts REEs more efficiently than Aliquat 336, especially from ethanolic and ethylene glycol solutions (Fig. 8). The addition of a PMOS increases the activity of the chloride anions, and hence enhances the ion-pair formation and extraction efficiency.^15,29^ However, solvent extraction with TODGA is not as selective for the (heavy) REEs as extraction chromatography with DGA resin. For instance, Fe(ii) is extracted quantitatively from ethylene glycol solution. The dilute nature of the feed solution in comparison with the capacity of the LP phase for metal ions is most likely the cause. If the extractant concentration or the volume phase ratio of the two immiscible phases would be lowered, the selectivity might enhance due to saturation effects. Increasing the water content in the ethylene glycol feed might also reduce co-extraction of contaminants.

## Conclusion

The effect of different polar molecular organic solvents on the retention of transition metals and rare-earth elements was studied for the anion exchanger Amberlite IRA 402 (chloride form) and two ion extraction chromatographic resins, TEVA (chloride form) and DGA, having Aliquat 336 and TODGA as the active functionality, respectively. The recovery of the metals was largely influenced by the dielectric constant of the feed solutions, as little to no retention of metal ions was observed from formamide or aqueous solutions (high dielectric constants), while enhanced sorption was observed in ethylene glycol and ethanol (low dielectric constants). The Amberlite IRA 402 resin was very efficient for recovery of Fe(ii), Cu(ii) and Co(ii) from 95 vol% ethanolic solutions, while the recovery of the rare-earth elements was limited, thus providing a method of removing transition-metal impurities from dilute rare-earth containing feeds. TEVA did not show affinity towards any metal ion for the tested organic solutions, due to a loss of functionality in the tested organic solvents (leaching of the active component from the solid support). The DGA resin recovered the rare earths with some selectivity, and is able to separate light from heavy rare-earth elements in ethylene glycol and ethanolic solution. However, the extractant incorporated in the resin (TODGA) was leached out at ethanol concentrations higher than 50 vol%, and hence the use of ethanol is not recommended in combination with DGA resin. Non-aqueous solvent extraction experiments using Aliquat 336 and TODGA were performed for comparison to the results of the ion exchanger and extraction chromatography resins. Volume changes were observed for the Aliquat 336 experiments, as well as for TODGA in combination with 50 vol% ethanol solutions. Ion exchange resins with covalent bonded functional groups, such as Amberlite IRA-402, do not display such losses to the feed solution, hence creating a clear advantage over non-aqueous solvent extraction. Impregnated resins, such as the TEVA and DGA resins, are often not compatible with organic solvents as these might leach out the extractants from the resin, and, therefore, are not recommended for use in non-aqueous ion exchange.

## Data availability

The data supporting this article have been included as part of the ESI.†

## Author contributions

Brecht Dewulf: conceptualisation, methodology, validation, formal analysis, investigation, writing – original draft, visualisation. Koen Binnemans: conceptualisation, writing – review & editing, supervision, resources, funding acquisition.

## Conflicts of interest

The authors declare that they have no known competing financial interests or personal relationships that could have appeared to influence the work reported in this paper.

## Supplementary Material

RA-015-D5RA00908A-s001
