# Supplementary material for: Effect of polar organic solvents on the separation of rare earths and transition metal chloride complexes: comparison of ion exchange, extraction chromatography and solvent extraction
Source: RSC Adv. 2025 Jun 3;15(23):18419–29. doi: 10.1039/d5ra00908a (PMC12131135; doi:10.1039/d5ra00908a)
Supplement: RA-015-D5RA00908A-s001 [file RA-015-D5RA00908A-s001.pdf]

Supplementary Information (SI) for RSC Advances.
This journal is © The Royal Society of Chemistry 2025

Electronic Supplementary Information (ESI)

Effect of polar organic solvents on the separation of rare earths and transition metal chloride complexes: comparison of ion exchange, extraction chromatography and solvent extraction

Brecht Dewulf†*, Koen Binnemans†

† KU Leuven, Department of Chemistry, Celestijnenlaan 200F, P.O. box 2404, B-3001 Leuven, Belgium.

*Corresponding author:

Email: brecht.dewulf@kuleuven.be

Calculation of dielectric constants

The dielectric constants of the mixtures were calculated in OLI Studio V11.5 using the mixed-solvent electrolyte framework (OLI Systems Inc., Parsippany NJ). Ethylene glycol was not available in the general mixed solvent electrolyte (MSE) database. Therefore, a new private OLI-MSE database was created to enable the prediction of the dielectric constant of mixtures of EG with water.[1] Adding only the thermodynamics properties of liquid EG suffices for this purpose. To this end, the standard state enthalpy of formation of liquid EG ($\Delta H_f^0(EG_{liq})$, -455.6 kJ mol^{-1}) was taken from Gardner *et al.*,[2] the standard state entropy of liquid EG ($S^0(EG_{liq})$, 166.9 J mol^{-1} K^{-1}) was taken from Parks *et al.*,[3] and the heat capacity (c_p, 149.3 J mol^{-1} K^{-1}) was used from Stephens *et al.*[4] The standard state Gibbs free energy of formation of liquid EG (ΔG_f^0, -323.92 kJ mol^{-1}) was calculated from $\Delta H_f^0(EG_{liq})$, $S^0(EG_{liq})$, and the sum of the entropy of elements that make up EG in their respective standard state (S^0(elements)), following equation S1:

$$\Delta G_f^0\left(EG_{liq}\right) = \Delta H_f^0\left(EG_{liq}\right) - T\left(S^0\left(EG_{liq}\right) - \sum_i S^0(element_i)\right) \qquad \text{(S1)}$$

The dielectric constant of the pure liquid as a function of the temperature was added the this database using an accurate fit to equation $\varepsilon = \varepsilon_0 + \varepsilon_1/T$ (temperature in kelvin). Based on data from the Dortmund Data Bank, ε_0 is -6.9096 and ε_1 is 13 564 in the range of 5 °C and 55 °C.[5] To accurately calculate the density, and thus the volume of solutions that contain EG, also the molar volume of pure liquid EG ($5.592 \cdot 10^{-5}$ m³ mol^{-1}) was added. This is based on the liquid density of EG (1.11 g mL^{-1}). Finally, to correct the entropy of EG due to mixing, size and surface parameters of the UNIQUAC framework were added.[6,7] They are 2.41 and 2.25, respectively.

---

[1] Wang, P.; Anderko, A. Computation of Dielectric Constants of Solvent Mixtures and Electrolyte Solutions. *Fluid Phase Equilib.* **2001**, *186* (1–2), 103–122. https://doi.org/10.1016/S0378-3812(01)00507-6.
[2] Gardner, P. J.; Hussain, K. S. The Standard Enthalpies of Formation of Some Aliphatic Diols. *J. Chem. Thermodyn*. **1972**, *4* (6), 819–827. https://doi.org/10.1016/0021-9614(72)90003-1.
[3] Parks, G. S.; Kelley, K. K.; Huffman, H. M. Thermal Data on Organic Compounds. V. A Revision of the Entropies and Free Energies of Nineteen Organic Compounds. *J. Am. Chem. Soc.* **1929**, *51* (7), 1969–1973. https://doi.org/10.1021/ja01382a003.
[4] Stephens, M. A.; Tamplin, W. S. Saturated Liquid Specific Heats of Ethylene Glycol Homologs. *J. Chem. Eng. Data* **1979**, *24* (2), 81–82. https://doi.org/10.1021/je60081a027.
[5] DDBST GmbH. *Dielectric Constant of 1,2-Ethanediol from Dortmund Data Bank*. http://www.ddbst.com/en/EED/PCP/DEC_C8.php (accessed 2023-08-18).
[6] Wang, P.; Anderko, A.; Young, R. D. A Speciation-Based Model for Mixed-Solvent Electrolyte Systems. *Fluid Phase Equilib.* **2002**, *203* (1–2), 141–176. https://doi.org/10.1016/S0378-3812(02)00178-4.
[7] Lancia, A.; Musmarra, D.; Pepe, F. Vapor-Liquid Equilibria for Mixtures of Ethylene Glycol, Propylene Glycol, and Water between 98 °C. and 122 °C. *J. Chem. Eng. Japan* **1996**, *29* (3), 449–455. https://doi.org/10.1252/jcej.29.449.

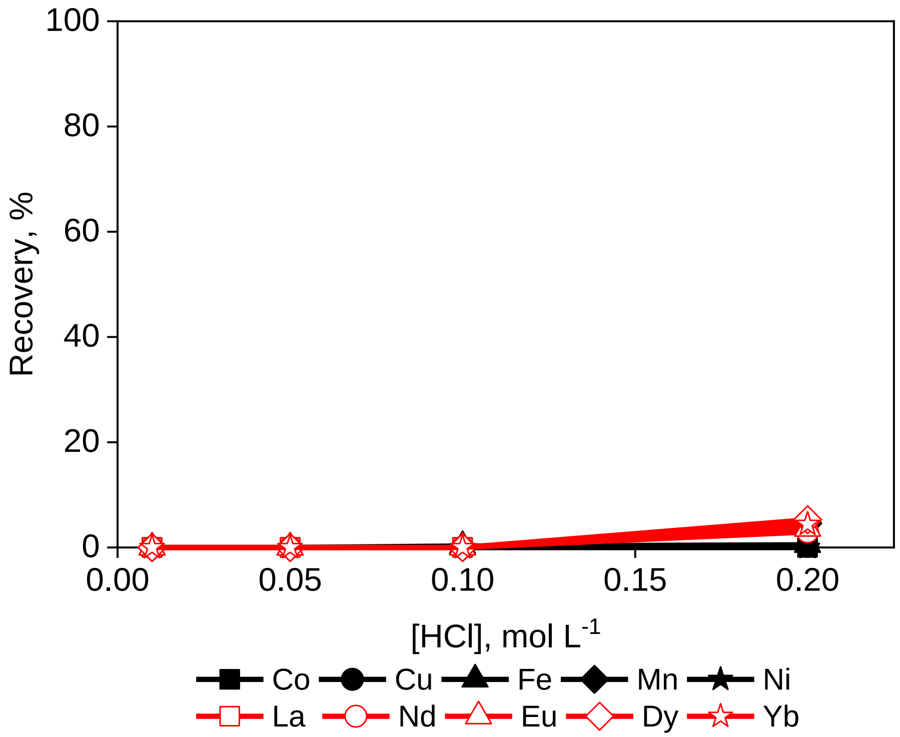

Figure S1. *Effect of HCl concentration on the sorption of transition metals and REEs from aqueous feeds by Amberlite IRA 402 (chloride form). Conditions: 25 mg of resin, 2.5 mL of feed solution, metal concentration 0.5 mmol L^{-1} (each), t = 30 min, T = 21 ± 1 °C.*

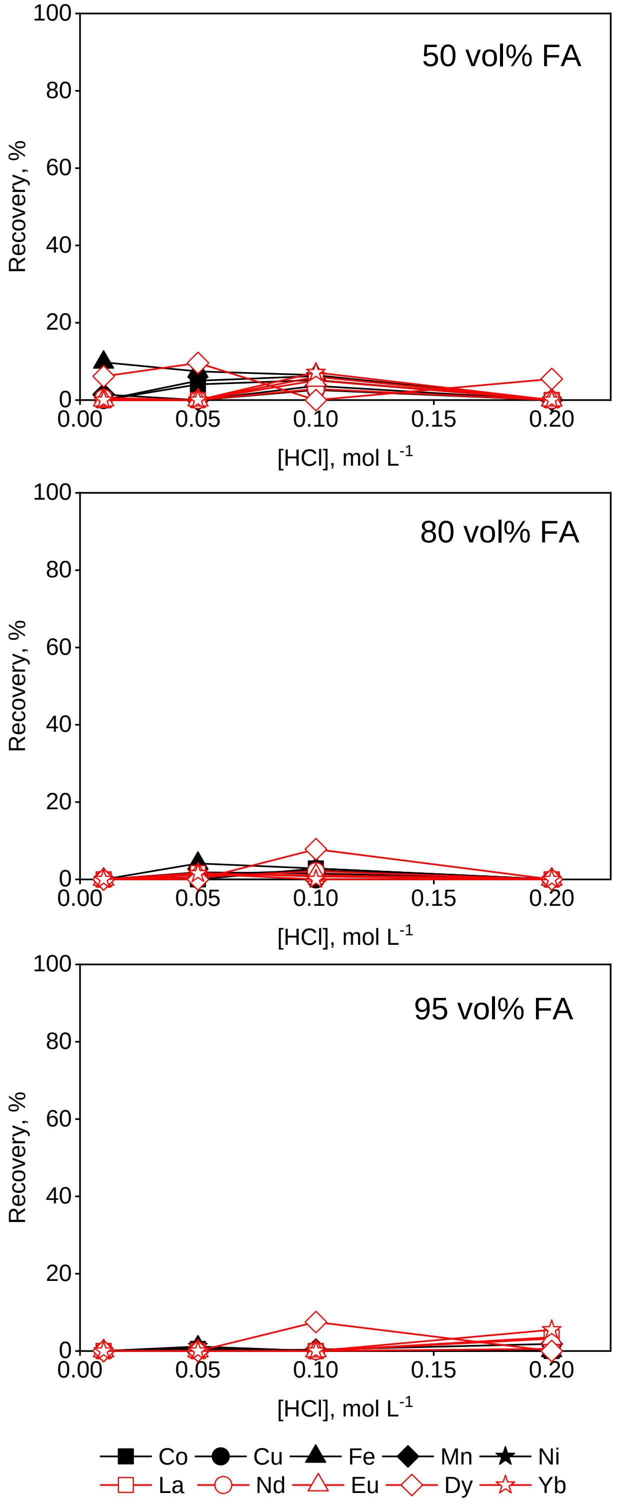

Figure S2. *Effect of HCl concentration of the sorption of transition metals and REEs from feeds containing 50, 80 or 95 vol% formamide (FA) by Amberlite IRA 402 (chloride form). Conditions: 25 mg of resin, 2.5 mL of feed solution, metal concentration 0.5 mmol L^{-1} (each), t = 30 min, T = 21 ± 1 °C.*

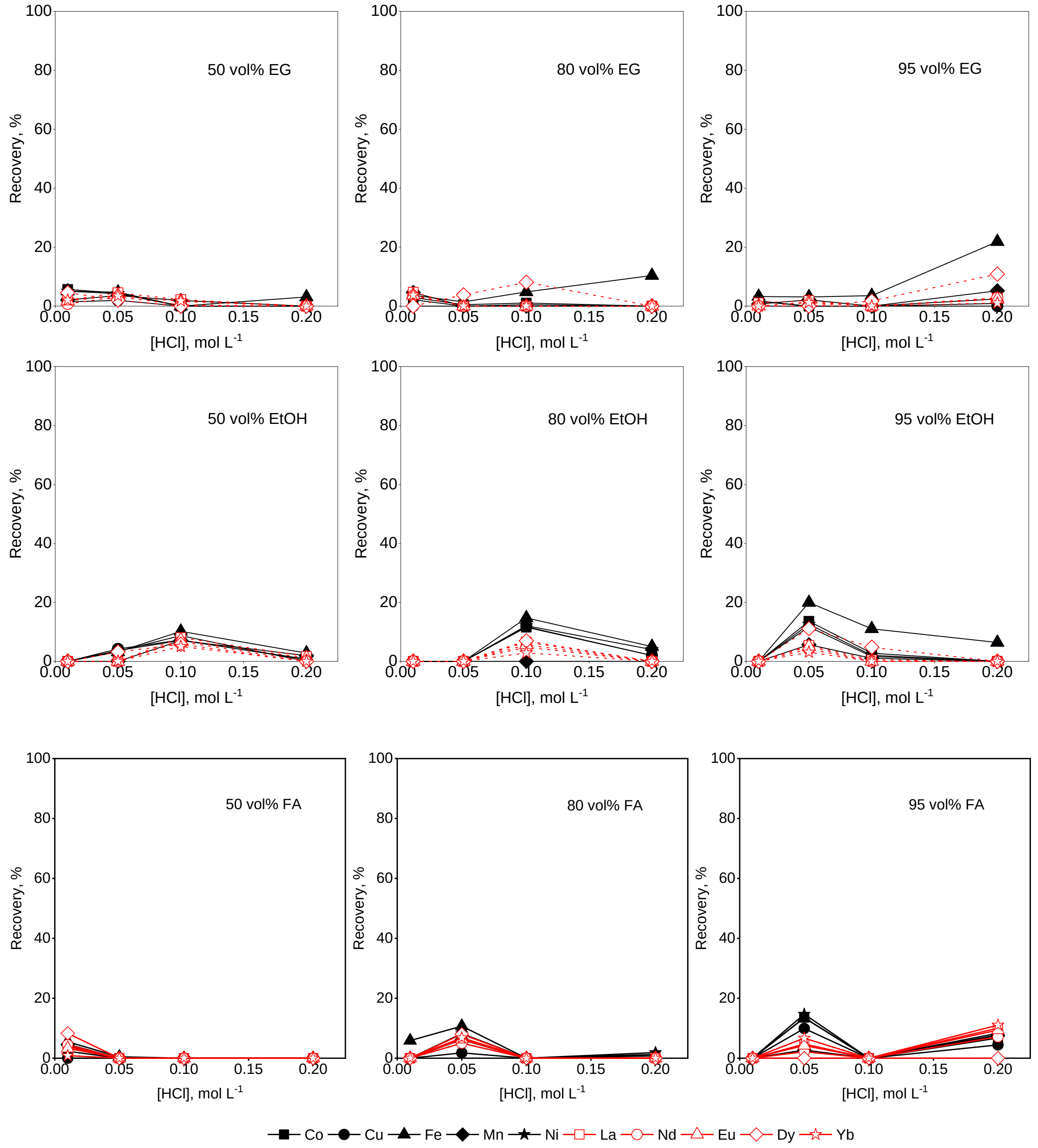

Figure S3. *Effect of HCl on the sorption of transition metals and REEs from ethylene glycol (EG), ethanol (EtOH) and formamide (FA) feeds (50, 80, 90 vol%) by TEVA (chloride form). Conditions: 25 mg of resin, 2.5 mL of feed solution, metal concentration 0.5 mmol L^{-1} (each), t = 30 min, T = 21 ± 1 °C.*

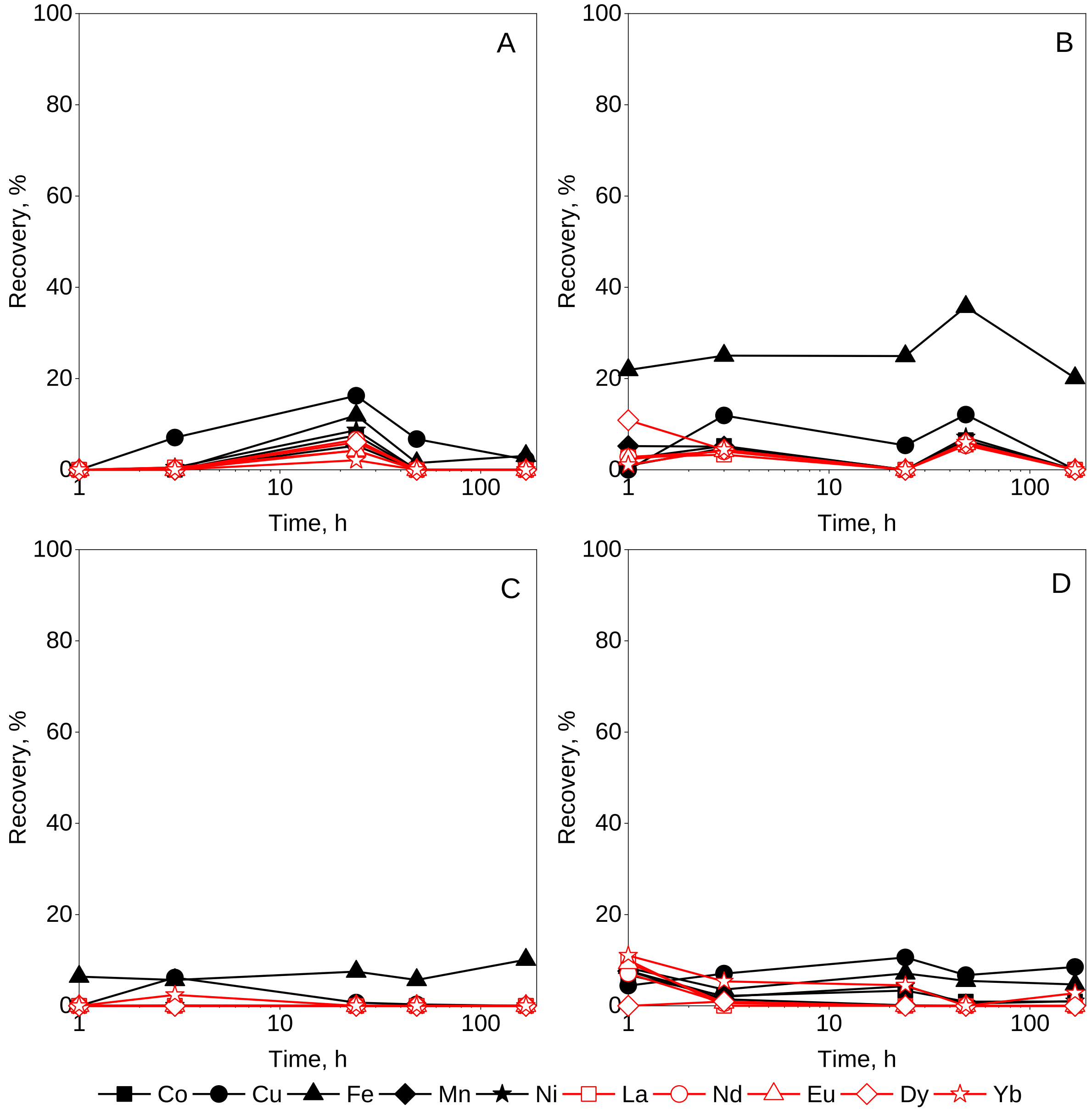

Figure S4. *Effect of time on the sorption of transition metals and REEs from (A) water, (B) 95 vol% ethylene glycol, (C) 95 vol% ethanol and (D) 95 vol% formamide feeds by TEVA (chloride). Conditions: 25 mg of resin, 2.5 mL of feed solution, metal concentration 0.5 mmol L^{-1} (each), 0.2 mol L^{-1} HCl, t = 30 min, T = 21 ± 1 °C.*

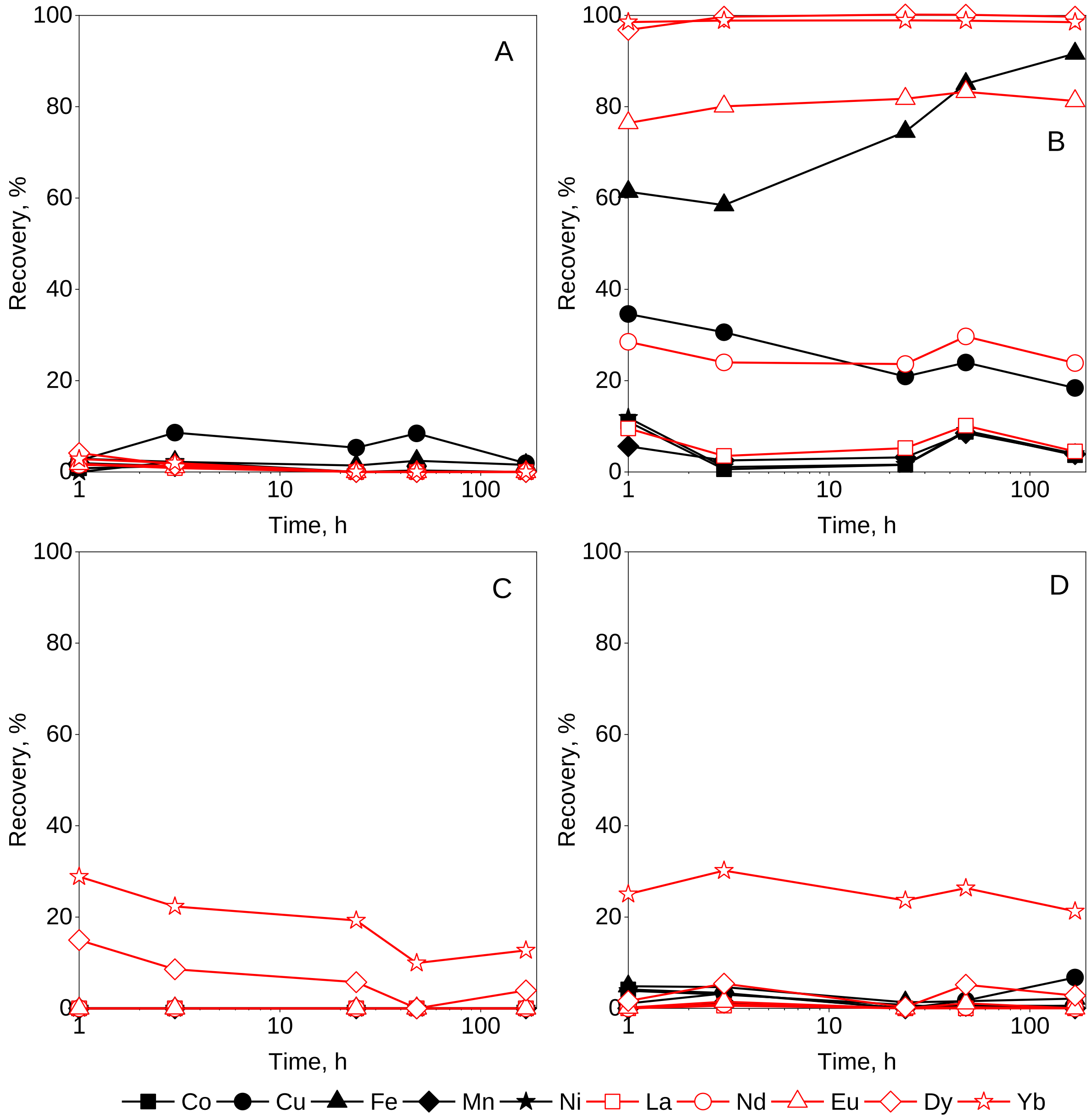

Figure S5. *Effect of time on the sorption of transition metals and REEs from (A) water, (B) 95 vol% ethylene glycol, (C) 95 vol% ethanol and (D) 95 vol% formamide feeds by DGA resin. Conditions: 25 mg of resin, 2.5 mL of feed solution, metal concentration 0.5 mmol L^{-1} (each), 0.2 mol L^{-1} HCl, t = 30 min, T = 21 ± 1 °C.*

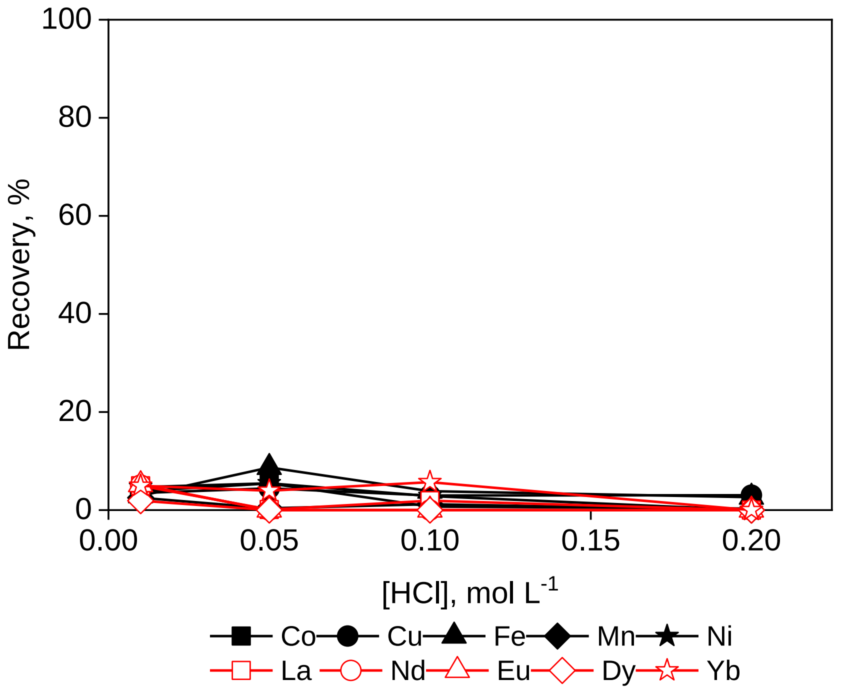

Figure S6. *Effect of HCl concentration on the sorption of transition metals and REEs from aqueous feeds by DGA chromatographic resin. Conditions: 25 mg of resin, 2.5 mL of feed solution, metal concentration 0.5 mmol L^{-1} (each), t = 30 min, T = 21 ± 1 °C.*

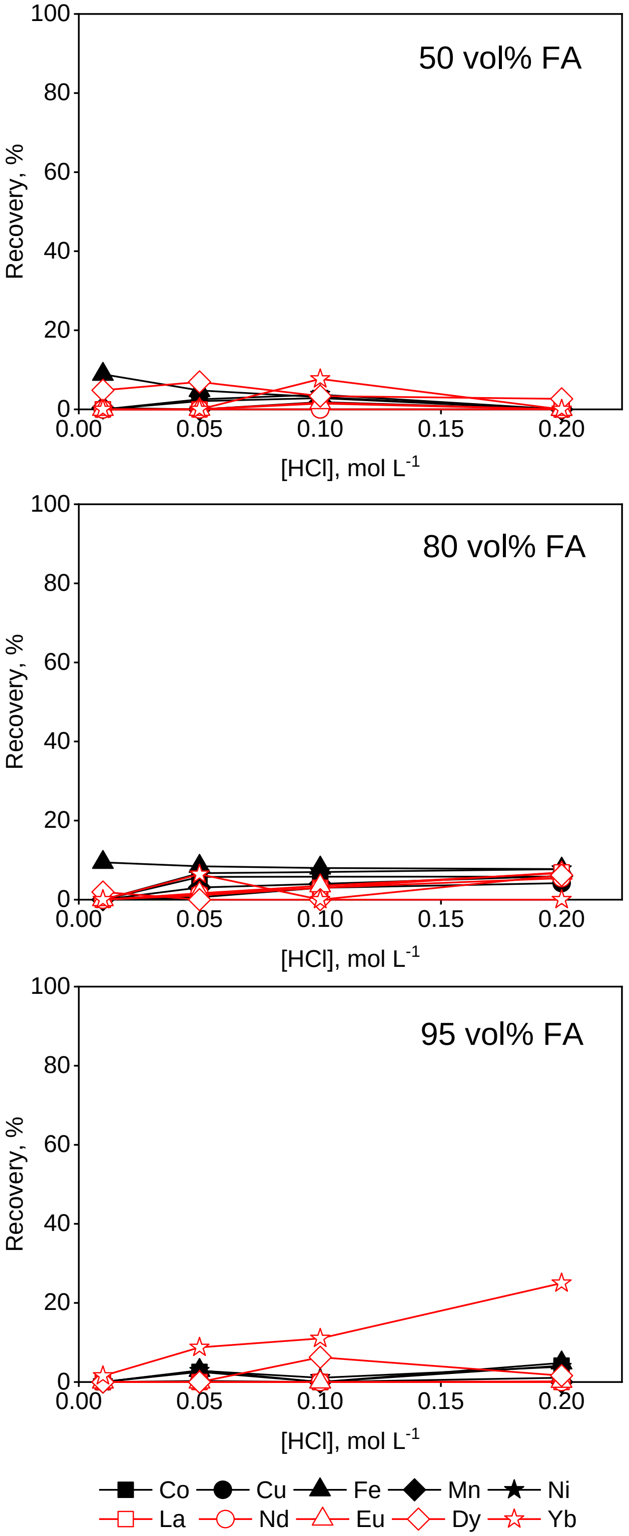

Figure S7. *Effect of HCl concentration of the sorption of transition metals and REEs from feeds containing 50, 80 or 95 vol% formamide (FA) by DGA chromatographic resin. Conditions: 25 mg of resin, 2.5 mL of feed solution, metal concentration 0.5 mmol L*$^{-1}$ *(each), t = 30 min, T = 21 ± 1 °C.*

Table S1. *Overview of the LP:MP ratios of the tested solvent extraction systems at equilibrium, in mL. Original phase ratio LP:MP = 4 mL : 4 mL.*

	**A336 – A150**			**TODGA – A150**			**TODGA – GS190**		
**[extractant], vol%**	**20**	**40**	**60**	**20**	**40**	**60**	**20**	**40**	**60**
**95 vol% EG**	4.1:3.9	4.3:3.7	4.6:3.4	4:4	4:4	4:4	4:4	4:4	4:4
**50 vol% EtOH**	5:3	5.2:2.8	5.5:2.5	4.2:3.8	4.4:3.6	4.5:3.5	4.2:3.8	4.4:3.6	4.5:3.5
**95 vol% FA**	Third phase		4.6:3.4	4:4	4:4	4:4	4:4	4:4	4:4
